# Evaluation of the Antioxidant Activity, Deodorizing Effect, and Antibacterial Activity of ‘Porotan’ Chestnut By-Products and Establishment of a Compound Paper

**DOI:** 10.3390/foods10051141

**Published:** 2021-05-20

**Authors:** Yoko Tsurunaga, Tetsuya Takahashi

**Affiliations:** Faculty of Human Science, Shimane University, Shimane 690-0823, Japan; takahashi@hmn.shimane-u.ac.jp

**Keywords:** ‘Porotan’ chestnut, *Castanea crenata* Sieb. et Zucc, antioxidant activity, deodorant activity, antibacterial activity, compound paper

## Abstract

Chestnuts are widely cultivated for their edible portion (kernel), whereas the non-edible parts are discarded. To enable the utilization of the by-products of processed chestnuts, we separated them into green and brown burs, shells, inner skin, and leaves, and analyzed the bioactive properties of the ground components. We also created a composite paper, comprising the inner skin, and examined its deodorant properties. It was revealed that the inner skin had the highest functionality and showed potent antioxidant, antibacterial, and deodorant properties. Furthermore, when we produced a paper, containing 60% inner skin, and examined its deodorant properties, we found that it was highly effective in deodorizing ammonia and acetic acid gases. These results show that the inner skin of chestnuts is a promising material for developing hygiene and other products.

## 1. Introduction

Chestnut cultivation and processing are performed worldwide. Chestnuts cultivated worldwide include the Japanese chestnut (*Castanea crenata* Sieb. et Zucc.), Chinese chestnut (*Castanea mollissima* Blume), and European chestnut (*C. sativa* Mill.) cultivated in Southern Europe and Asia Minor. According to the data from the Food and Agriculture Organization, Asia is the leading global producer of chestnuts (86.5%), followed by Europe (9.6%) and the United States (3.8%) [1]. The kernel (edible part) of chestnuts is used to prepare glacé, pastes, and syrups [2]; however, additional parts of the chestnut (*Castanea crenata* Sieb. et Zucc.), including the leaves, burs, shells, and the inner skin, are currently not used. Chestnut shells contain various phenolic compounds [3], and the tough skin contains a large amount of tannins [4], which show antioxidant [3,5,6], fat absorption-inhibiting [7], and anti-adipogenic activities. Tannins also reportedly have preventive effects against heart disease [8], diabetes [9], and cancer [10], as well as antibacterial [11,12] and deodorizing properties [13]; therefore, the utilization of fruit peels is considered important from an industrial perspective [14]. However, the existing Japanese varieties of chestnuts are difficult to peel, and the bioactivity of the inner bark of trees have not been investigated. The shell and inner skin of the variety ‘Porotan’, cultivated in Japan, can be peeled extremely well [15,16]; therefore, the inner skin can be used as a by-product after the processing of chestnut kernels. In this study, we aimed to utilize the unused parts of chestnuts for food and industrial applications and analyzed the functionality (polyphenol content, antioxidant activity, anti-bacterial activity and deodorizing activity) of the chestnut burs (green and brown), leaves, shells, inner skin, and kernel. Secondly, we tested the functionality (deodorizing activity) of the produced compound paper, which contains 60% inner skin. It was clarified that the inner skin had the highest polyphenol content, antioxidant, anti-bacterial, and deodorant properties. Further, we found that the paper containing 60% inner skin is highly effective in deodorizing ammonia and acetic acid gases. This research will lead to the effective utilization of the large amounts of by-products generated from chestnut cultivation and processing.

## 2. Materials and Methods

### 2.1. Samples

#### 2.1.1. Japanese Chestnut ‘Porotan’ 

**Figure 1:**
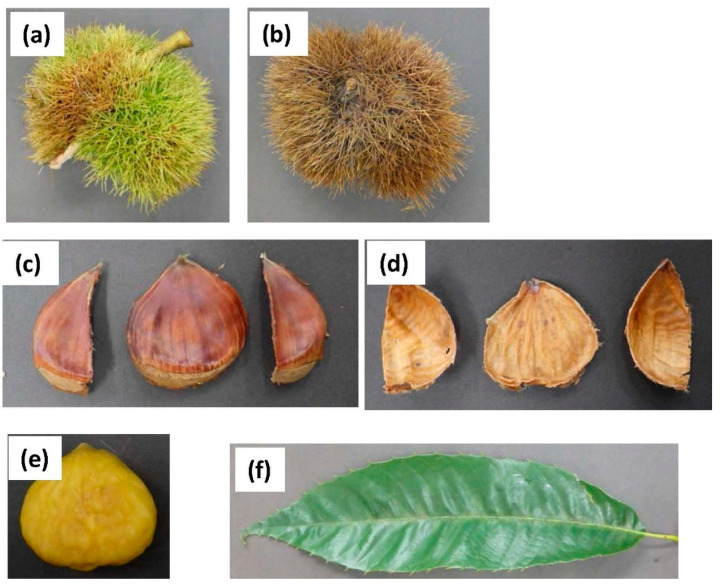
Photographs of Japanese chestnut ‘Porotan’ components. (**a**): Burs (green), (**b**): burs (brown), (**c**): shells, (**d**): inner skin, (**e**): kernel, (**f**): leaf.

Burs (green and brown), shells, inner skin, kernel, and leaves were obtained from Japanese chestnut ‘Porotan’ trees (scientific name: *Castanea crenata* Sieb. et Zucc.) in the Institute of Fruit Tree Science of Ibaraki Prefecture, Japan, during September 2017 (Figure 1). The materials were freeze-dried and ground into a powder for subsequent analyses. The powder was ground in a blender for approximately 120 s, and then sieved through a mesh sieve (1 mm) to obtain samples.

#### 2.1.2. Effects of Chestnut Samples on Bacteria

*S. aureus* NBRC 12732 and *E. coli* NBRC 3301 were obtained from the National Institute of Technology and Evaluation, Osaka, Japan, and used to analyze the antibacterial activities of leaves, burs (green and brown), shells, and inner skin in ‘Porotan’ trees.

#### 2.1.3. Preparation of Compound Papers

Compound papers were prepared using a previously reported method [17,18,19,20]. 

### 2.2. Analysis of Properties

#### 2.2.1. Extraction Method for Total Polyphenol Content (TPC), 2,2-Diphenyl-1-Picrylhydrazyl (DPPH), and Hydrophilic Oxygen Radical Absorbance Capacity (H-ORAC) Assays

The prepared powdered samples (200 mg) were added to 60% (*v*/*v*) ethanol aqueous (10 mL) and shook for 2 h at 40 °C. The extracted was filtered through a 0.45 µm filter.

#### 2.2.2. TPC

The soluble polyphenol content of the extracts was measured by the Folin–Ciocalteu method [21]. The TPC was expressed as mg equivalent/100 g of dry matter, using catechin (CTN) as the standard (mg CTN eq/100 g DW).

#### 2.2.3. DPPH Radical Scavenging Activity Assay

The antioxidant activity of the extracts was analyzed using the DPPH radical scavenging assay [22]. The DPPH value was expressed as µmol Trolox equivalent/g of dry matter (µmol TE/g DW).

#### 2.2.4. Hydrophilic-ORAC (H-ORAC)

The antioxidant activity of the extracts was analyzed using the H-ORAC method [20,23]. H-ORAC values were expressed as Trolox equivalents of dry matter (μmol TE/g DW).

#### 2.2.5. Antibacterial Properties

Antibacterial activity assays were performed by modifying methods by which we had previously determined the antibacterial activity [17,18,19,20,24]. Using these methods, the activity of each part of Porotan was examined, and all parts except for kernel showed very strong activity (a kill rate of 100%) with no differences. To clarify the difference in antibacterial properties in each part of Porotan, the experiment was performed by applying stricter conditions to the previous method [17,18,19,20,24]. Specifically, we increased the amount of a bacterial suspension from 100 to 200 µL, the sample amount was reduced from 200 to 40 mg. The analyzed parts included the burs (green and brown), shells, inner skin, and leaves, based on the results of antioxidant activities. For comparison, green tea, which reportedly contains potent antibacterial properties was used. 

#### 2.2.6. Evaluation of Deodorant Activity

Deodorant activity assays were performed by the modifying methods that we had previously determined [19,20,25]. The odor residual ratio was plotted against time. The odor residual ratio of the sample was calculated using Equation (1) [19,20,25].

(1) $$\mathrm{Odor}\;\mathrm{residual}\;\mathrm{ratio}\;(\%)=\frac{\mathrm{Measured}\;\mathrm{gas}\;\mathrm{concentration}}{\mathrm{Initial}\;\mathrm{concentration}}\times 100$$

#### 2.2.7. Scanning Electron Microscopy

The fixed samples were dried at room temperature for 24 h, and then gold was vapor-deposited on the sample using an ion sputterer (E-1010, Hitachi High-Technologies Corp., Tokyo, Japan). The surface of the inner skin of ‘Porotan’ chestnuts was observed using a scanning electron microscope S-3000N (Hitachi Science Systems, Ltd., Tokyo, Japan). The microscope was operated at an acceleration voltage of 20 kV.

#### 2.2.8. Statistical Analysis

Data from TPC, DPPH, and H-ORAC assays were statistically analyzed using the SPSS statistical analysis software (version 25.0, SPSS Inc., Chicago, IL, USA). Results are expressed as the mean ± standard error. Data were tested using one-way-ANOVA, followed by Tukey’s test for multiple comparisons.

## 3. Results and Discussion

### 3.1. TPC and Antioxidant Activity

**Figure 2:**
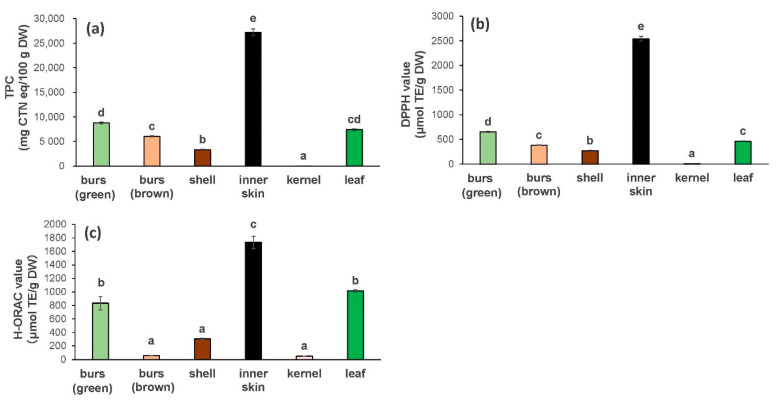
Antioxidant activity of ‘Porotan’ chestnut components. Antioxidant activity was evaluated as the TPC (**a**), DPPH radical scavenging activity (**b**), and H-ORAC (**c**). TPC, total polyphenol content; DPPH, 2,2-diphenyl-1-picrylhydrazyl radical scavenging activity; H-ORAC, hydrophilic oxygen radical absorbance capacity. Means are shown, with vertical bars indicating standard error (*n* = 6). The different letters (a–e) indicate statistical differences (*p* < 0.05).

Figure 2 shows the TPC and antioxidant activity of each of the following parts of the Porotan: burs (green and brown), shells, inner skin, and leaves. The TPCs in the green burs, brown burs, shells, inner skin, kernels, and leaves were 8770 ± 235, 6047 ± 125, 3325 ± 110, 27,208 ± 110, 12 ± 4.5, and 7414 ± 161 mg CTN eq/100 g DW, respectively. The antioxidant activities (DPPH) of the green burs, brown burs, shells, inner skin, kernels, and leaves were 653 ± 9.5, 384 ± 4.1, 269 ± 12.0, 2452 ± 45.0, 7.0 ± 1.0, and 461 ± 7.4 μmol TE/g DW, respectively. The antioxidant activities (H-ORAC) of the green burs, brown burs, shells, inner skin, kernels, and leaves were 833 ± 99.3, 55.3 ± 0.3, 304.3 ± 12.5, 1733 ± 91.2, 50 ± 0.8, and 1017 ± 16.0 μmol TE/g DW, respectively. The TPC and antioxidant activities (H-ORAC and DPPH method) of all parts of the chestnut, except for the kernel, were very high; the inner skin showed the highest TPC and antioxidant activity. Tuyen et al. [26] evaluated the antioxidant activity of the Japanese chestnut (barks, flowers, inner skin, kernels, and leaves) and reported that the inner skin showed the highest antioxidant activity in all the assays used (DPPH, 2,2-azinobis [3-ethylbenzothiazoline-6-sulfonic acid] diammonium salt, reducing power, and β-carotene bleaching methods). These findings are consistent with our results, which showed that the inner skin had the highest DPPH radical scavenging activity. In addition, we revealed that the inner skin had a high level of antioxidant activity with the H-ORAC method, which offers several advantages over the DPPH assays. The complete hydrogen atom transfer radical quenching mechanism presents a contrast and comparison to the electron transfer in the DPPH assays [27]. The ORAC assay uses peroxyl radicals that present a better model of the antioxidation reaction between oxidative lipids and reactive oxygen species [27]. The high values obtained via the ORAC assay are of great importance in terms of health functionality. Chestnut shells contain high concentrations of ellagic acid, a phenolic compound [5]. Ellagic acid is a dimeric derivative of gallic acid [28,29] and has certain health functions with specific biological effects, such as anti-angiogenesis [30], anti-cancer [10], anti-diabetes [31], and antioxidant [32]. Chestnut shells are expected to be used as highly valuable materials in the future. Additionally, we also found strong activity for burs, which Tuyen et al. [26] did not measure. The burs are a part of the plant that is removed during harvesting rather than peeling, thereby making it feasible to collect. In this study, we found that the green burs had higher TPC, DPPH, and H-ORAC values than the brown burs. Notably, the green burs had an H-ORAC value of 833 μmol TE/g DW, whereas that of the brown burs was 55.3 μmol TE/g DW, which was considerably low. Therefore, the inner skin, leaves, and burs (green) may present promising materials with antioxidant activity.

### 3.2. Antibacterial Activity

Gram-positive *S. aureus* and Gram-negative *E. coli* were used to assess the antibacterial activity of each part of the chestnut. The results are shown in Table 1. As a result, the growth values of both bacterial species yielded were above the JIS standard value for the assay of 0.5. Therefore, both of the species showed growth without inhibition, in the antibacterial assays. *S. aureus* showed growth from a viable count of 1.00 × 10^5^ CFU/mL before incubation, to 1.04 × 10^10^ CFU/mL after 18 h of incubation in the culture medium without chestnut samples (control); therefore, the growth was increased by approximately 10^5^-fold. In contrast, when cultured with the inner skin of the chestnuts, the viable count increased to 2.04 × 10^5^ CFU/mL after incubation. Considering the viable count of 1.04 × 10^10^ CFU/mL of the control, the antibacterial effect corresponded to an approximately 10^5^-fold decrease in cell count. In this experiment, we modified our previous methods, the amount of bacterial cell suspension was increased from 100 to 200 µL [17,18,19,20,24], the sample amount was reduced from 200 to 40 mg [17,18,19,24], and the experiment was conducted under conditions that enhanced bacterial growth. Therefore, the cultures incubated with green tea [33], which has high antibacterial activity, produced a bacterial count of 1.80 × 10^9^ CFU/mL. This indicated that the inner skin might be highly effective in inhibiting *S. aureus* growth. The cultures incubated with green burs, brown burs, shells, and leaves showed a viable count of 3.84 × 10^6^, 5.80 × 10^6^, 1.36 × 10^6^, and 1.16 × 10^6^ CFU/mL, respectively, and thus, showed higher antibacterial activity than green tea. *E. coli* showed growth from a viable count of 1.00 × 10^5^ CFU/mL before incubation to 2.80 × 10^9^ CFU/mL after 18 h of incubation without chestnut samples (control); therefore, the growth was increased by approximately 10^5^-fold. When the cultures were incubated with brown burs, the viable count increased to 2.28 × 10^7^ CFU/mL after incubation. Considering the viable count of 2.8 × 10^9^ CFU/mL of the control, the antibacterial effect corresponded to an approximately 100-fold decrease in cell count. Similar to the experiment using *S. aureus*, the amount of the bacterial cell suspension was increased from 100 to 200 µL, the sample amount was reduced from 200 to 40 mg, and conditions that enhanced bacterial growth were used. Therefore, the cultures incubated with green tea [33] produced a bacterial count of 4.64 × 10^8^ CFU/mL. The cultures incubated with green burs, brown burs, shells, inner skin and leaves showed a viable count of 4.40 × 10^8^, 2.28 × 10^7^, 2.28 × 10^10^, 2.16 × 10^8^, and 1.24 × 10^9^ CFU/mL, respectively. Only the brown burs had an antibacterial activity value of over 2, which meant that it reduced the number of cells by a factor of 100, and showed a higher antibacterial effect than green tea. This indicated that brown burs might be highly effective in inhibiting *E. coli* growth. *S. aureus* is a Gram-positive bacterium that contains a thick cell wall layer, mostly consisting of peptidoglycan, whereas the cell wall of the Gram-negative *E. coli* contains a small quantity of peptidoglycan. Generally, Gram-positive bacteria contain a peptidoglycan layer as thick as 20–80 nm, while Gram-negative bacteria contain a layer that is 7–8 nm thick; therefore, Gram-positive bacteria have a considerably thicker cell wall than that of Gram-negative bacteria. The DW of the bacterial cell wall is approximately 10% in Gram-negative bacteria. By contrast, the Gram-positive bacteria weigh as much as 90%. Based on the above results, it was predicted that *E. coli*, a Gram-negative bacterium, would show a higher inhibition of growth than *S. aureus* when treated with chestnut components; however, a different result was obtained. It has been reported that tea polyphenols show a higher antibacterial activity against *S. aureus* than against *E. coli* [33,34,35]; therefore, the result may not be explained by the thickness of the cell wall alone. The high antimicrobial activity of tea is due to the existence of catechins and polyphenols that damage the cell membranes of bacteria [36]. Catechins are more active against Gram-positive bacteria than Gram-negative bacteria [35]. EGCg inhibits cell membrane function by eliciting the leakage of small molecules from the intraliposomal space [35]. Porotan, like green tea, has a bacterial membrane-damaging effect, and this effect was more pronounced in *S. aureus* than in *E. coli*. JIS-L-1902 [37] specifies that an assay value of antibacterial activity of 2.0 or higher (a kill ratio of 99% or higher) indicates an antibacterial effect. The antibacterial activity of green and brown burs, shells, inner skin, and leaves against *S. aureus*, and that of brown burs against *E. coli*, met the criteria, indicating that they might act as potent antimicrobial materials. Each part of ‘Porotan’ was found to have a stronger antibacterial effect against *S. aureus* than against *E. coli*.

### 3.3. Deodorizing Activity of the Inner Chestnut Skin and Compound Paper

**Figure 3:**
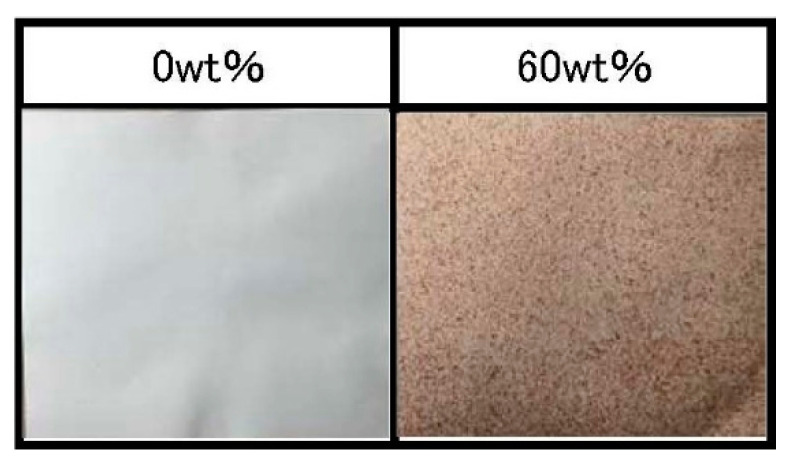
Compound paper (25 × 25 cm) comprising the inner skin of ‘Porotan’ chestnuts (60% weight).

**Figure 4:**
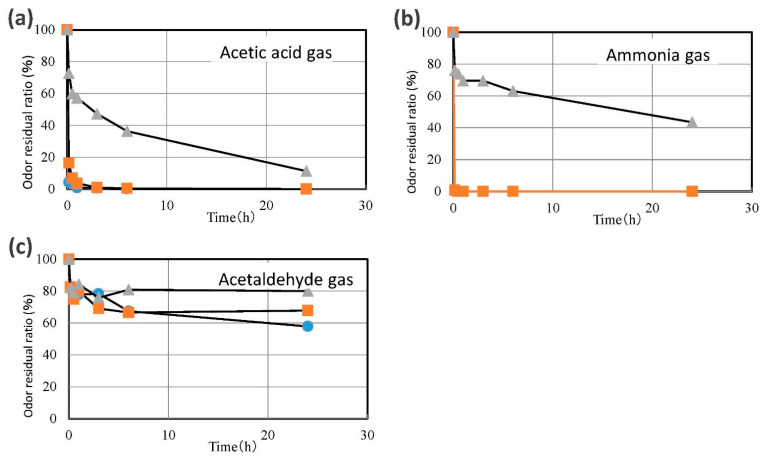
Deodorizing effects of the inner skin of ‘Porotan’ chestnuts and compound papers against various odorous gases. (**a**) Acetic acid gas, (**b**) ammonia gas, (**c**) acetaldehyde gas. The compounded papers were prepared by blending the ground inner skin of chestnuts (60% weight) with pulp fibers. ●, compound papers; ∎, inner skin; ▴, no sample against gas (control).

**Figure 5:**
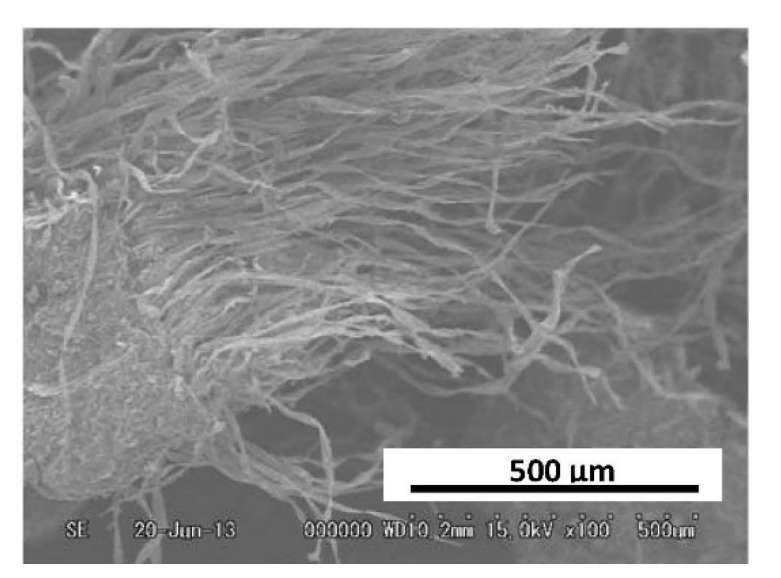
Scanning electron micrographs of skin of ‘Porotan’ (×500). Acceleration voltage: 20 kV.

The inner skin, which indicated high antioxidant and antibacterial activities, was selected for analysis of the deodorizing activity against various kinds of odors. The odorous gases used in the deodorization assays were ammonia (a basic odor), acetic acid (acidic odor), and acetaldehyde (neutral odor). We additionally analyzed the deodorizing properties of a compound paper containing 60% of inner chestnut skin (Figure 3). The change in odor residual ratio was found to be time-dependent (Figure 4). The results revealed that the inner skin and the compound paper effectively deodorized the basic odor of ammonia and the acidic odor of acetic acid. The odor residual ratio of ammonia gas decreased to 0% in just 10 min. The potent activity should be evaluated in further studies. The mechanism of the deodorization of ammonia gas by the inner skin and compound paper was considered to be that the hydroxyl groups of the polyphenols in the inner skin form hydrogen bonds with the ammonia molecules. In summary, certain OH groups of the polyphenols in the inner skin might have been converted into O–NH_4_^+^ to incorporate ammonia gas into their molecular structures [19]. Additionally, the odor residual ratio for acetic acid gas incubated with the inner skin and compound paper was found to considerably decrease to 7.0 and 3.1% in 30 min, respectively. Therefore, the inner skin and compound paper were found to have a high deodorization capacity against acidic odor. The Weber–Fechner law indicates that the sensory intensity of an odor is in proportion to the logarithm of the odorant concentration. Therefore, when the concentration of an odor increases by 10-fold, the intensity of the odor can be perceived as two-fold. Conversely, the odor of one-tenth of the concentration may be perceived as 50% as intense as that of the original concentration. In conclusion, the ground inner skin and the compound paper produced using the skin were found to decrease the sensory intensity of the basic and acidic odors by approximately 50%. The inner skin of the chestnut showed a unique fibrous shape (Figure 5). Similarly, charcoal shows a high porosity, which is associated with its deodorizing effect [38]. The porosity of the inner skin could also be associated with its deodorizing effect. In contrast, the ground inner skin and compound paper showed negligible deodorizing activity toward acetaldehyde, a neutral odor. Further studies should be performed to determine the differences in the deodorizing effects of the inner chestnut skin and compound paper on different odor components.

## 4. Conclusions

To determine the potential re-utilization of the by-products of processed chestnut, chestnuts were separated into green and brown burs, shells, inner skin, kernel, and leaves; each part was powdered for analysis of its bioactive properties. Compound paper comprising inner skin was prepared, and its deodorizing properties were investigated. The analysis of the TPC, DPPH, and H-ORAC values showed that the inner skin possessed high antioxidant activity. Ground inner skin, green and brown burs, shells, and leaves showed excellent antibacterial activity against *S. aureus*, and brown burs showed antibacterial activity against *E. coli*. The inner skin considerably inhibited the growth of Gram-positive *S. aureus* and was found to possess high deodorant effect against basic and acidic odors. It also decreased the odor residual ratio of ammonia gas to 0% in only 10 min. A compound paper was prepared, comprising the inner skin of chestnuts, and it was found to possess a high deodorant effect. These results indicate that the inedible part of chestnuts has a very high potential for use in food and industrial products, and it is expected to be used in the future.

## Figures and Tables

**Table 1:** Antibacterial properties of ‘Porotan’ chestnuts against different bacteria.

Species of Bacterium	Sample	Incubation Time (h)	Antibacterial Properties		Bacteria Yielded Growth Values *^3^
			Viable Bacteria (CFU *^1^/mL)	Antibacterial Activity Value *^2^	
*Staphylococcus aureus*	Before incubation	0	1.00 × 10^5^	−	
	Burs (green)	18	3.84 × 10^6^	3.43	
	Burs (brown)	18	5.80 × 10^6^	3.25	
	Shell	18	1.36 × 10^6^	3.21	
	Inner skin	18	2.04 × 10^5^	4.03	
	Leaf	18	1.16 × 10^6^	3.28	
	Green tea	18	1.80 × 10^9^	0.76	
	Only bacteria	18	1.04 × 10^10^	−	5.02
*Escherichia coli*	Before incubation	0	1.00 × 10^5^	−	
	Burs (green)	18	4.40 × 10^8^	0.80	
	Burs (brown)	18	2.28 × 10^7^	2.09	
	Shell	18	2.28 × 10^10^	−0.91	
	Inner skin	18	2.16 × 10^8^	1.11	
	Leaf	18	1.24 × 10^9^	0.35	
	Green tea	18	4.64 × 10^8^	0.78	
	Only bacteria	18	2.80 × 10^9^	−	4.45

*^1^: Colony-forming units. *^2^: Antibacterial activity value = (logC_t_ − logC_0_) − (logT_t_ − logT_0_). logCt: log (Ave-age (count of viable cell after incubation of bacteria alone for a given period)); logC_0_: log (average (count of viable cell before incubation)); logT_t:_ log (average (count of viable cell after incubation with chestnut samples for a given period)); logT_0:_ log (average (count of viable cell of the samples immediately after inoculation)). *^3^: Bacteria yield growth values = logC_t_ − logC_0_.
